# Barriers and facilitators of reproductive autonomy of adolescent girls in western Uganda: a qualitative inquiry

**DOI:** 10.1080/26410397.2026.2683272

**Published:** 2026-06-08

**Authors:** Linda Grace Alanyo, Sofie Vindevogel, Emmanuel Kimera, Lynn M Atuyambe, Johan Bilsen

**Affiliations:** a Lecturer, Department of Nursing and Midwifery, Mountains of the Moon University, Fort Portal, Uganda; Doctoral Researcher, Department of Public Health, Mental Health and Wellbeing Research Group, Vrije Universiteit Brussel, Brussels, Belgium; Researcher, Centre for Health Research and Implementation Science, Uganda.; b Lecturer, Social Work & E-QUAL, Faculty of Education, Health, University of Applied Sciences and Arts, Gent, Belgium; c Researcher, Department of Public Health, Mental Health and Wellbeing Research Group, Vrije Universiteit Brussel, Brussels, Belgium; Researcher, Centre for Health Research and Implementation Science, Uganda; Senior Lecturer, Department of Public Health, Mountains of the Moon University, Fort Portal, Uganda; d Associate Professor, Department of Community Health and Behavioral Sciences, School of Public Health, Makerere University, Kampala, Uganda; e Professor, Department of Public Health, Mental Health and Wellbeing Research Group, Vrije Universiteit Brussel, Brussels, Belgium

**Keywords:** adolescent, qualitative, reproductive autonomy, reproductive health

## Abstract

Reproductive autonomy, the power to make decisions about and to control matters related to contraceptive use, pregnancy, and childbirth, is essential for women to attain their sexual and reproductive health and rights. We explored barriers and facilitators of reproductive autonomy among adolescent girls in western Uganda, a region marked by high rates of adolescent pregnancy and HIV prevalence. This qualitative inquiry involved 31 in-school and out-of-school adolescent girls aged 15–19 years, purposively selected from diverse communities within Fort Portal city, western Uganda. Data were collected through individual interviews between September and November 2024 and analysed using framework thematic analysis guided by the three dimensions of reproductive autonomy: decision-making, communication, and freedom from coercion. The findings show an intricate interaction of individual, relational, and structural factors that shape adolescent girls’ reproductive autonomy, with commonalities and uniqueness based on schooling status. Key barriers included control by parents and partners in communication and decision-making, limited knowledge of girls on sexual and reproductive health, low self-efficacy to decide and implement decisions, financial dependence, and lack of life basics. Conversely, supportive relationships and schooling emerged as critical facilitators. In conclusion, enhancing reproductive autonomy requires multi-level and multi-faceted interventions to address the barriers while leveraging the facilitators. These should include household economic empowerment, parenting support programmes, and policies that promote universal access to education. *DOI: 10.1080/26410397.2026.2683272*

## Plain language summary

We studied what helps and what makes it harder for adolescent girls in western Uganda to make their own free choices about using contraceptives, engaging in sex, getting pregnant, and giving birth. We held interviews with 31 adolescent girls in the age range of 15–19 years from September to November 2024. Eleven girls were attending school while 20 were not. These girls were selected from different communities in Fort Portal city, western Uganda. We found a number of issues that affected these girls’ ability to make decisions about their Sexual and Reproductive Health (SRH). Some of the main challenges were strong influence from parents and sexual partners, limited knowledge of SRH, low confidence to decide and implement decisions, being financially dependent on parents and sexual partners, and lack of basic needs. On the other hand, being in school and having supportive people, such as family members, around the girls helped them to feel more in control and able to decide on SRH matters. To help these girls have more say over their SRH, the findings emphasise efforts at different levels of society and from different people with whom they regularly interact. Such efforts could include helping families improve their incomes to financially support the girls, supporting parents on how to communicate with the girls, and making sure all girls can get education.

## Résumé

L’autonomie reproductive, c’est-à-dire le pouvoir de prendre des décisions et de maîtriser les questions relatives à la contraception, à la grossesse et à l’accouchement, est essentielle pour la santé et les droits sexuels et reproductifs des femmes. Nous avons étudié les obstacles et les facteurs favorisant l’autonomie reproductive chez les adolescentes dans l’ouest de l’Ouganda, une région marquée par des taux élevés de grossesse chez les adolescentes et de prévalence du VIH. Cette étude qualitative a porté sur 31 adolescentes, scolarisées ou non, âgées de 15 à 19 ans, sélectionnées à dessein dans plusieurs communautés de la ville de Fort Portal, dans l’ouest de l’Ouganda. Les données ont été recueillies au cours d’entretiens individuels entre septembre et novembre 2024, puis examinées à l’aide d’une analyse thématique cadrée, guidée par les trois dimensions de l’autonomie reproductive: la prise de décision, la communication et l’absence de coercition. Les résultats montrent une interaction complexe de facteurs individuels, relationnels et structurels qui façonnent l’autonomie reproductive des adolescentes, avec des points communs et des spécificités selon leur statut scolaire. Les principaux obstacles comprenaient la domination des parents et des partenaires dans la communication et la prise de décision, les connaissances limitées des jeunes filles en matière de santé sexuelle et reproductive, leur faible capacité à décider et à mettre en œuvre des décisions, leur dépendance financière et le manque de ressources de base. À l’inverse, les relations de soutien et la scolarisation sont apparues comme des facteurs facilitateurs essentiels. En conclusion, l’amélioration de l’autonomie reproductive exige des interventions à plusieurs niveaux et à plusieurs volets pour lever les obstacles tout en tirant parti des facteurs facilitateurs. Ces interventions devraient inclure l’autonomisation économique des ménages, des programmes de soutien aux parents et des politiques qui favorisent l’accès universel à l’éducation.

## Resumen

La autonomía reproductiva (AR), el poder para tomar decisiones sobre cuestiones relacionadas con el uso de métodos anticonceptivos, el embarazo y el parto, y controlarlas, es esencial para que las mujeres puedan realizar su salud y derechos sexuales y reproductivos. Exploramos los obstáculos y facilitadores de AR entre las adolescentes en el oeste de Uganda, una región marcada por altas tasas de embarazo en la adolescencia y prevalencia de VIH. Esta investigación cualitativa involucró a 31 adolescentes de 15 a 19 años, en la escuela y fuera de la escuela, seleccionadas intencionalmente de diversas comunidades de la ciudad Fort Portal, en el oeste de Uganda. Entre septiembre y noviembre de 2024, se realizaron entrevistas individuales para recolectar datos, que fueron analizados utilizando el análisis temático guiado por las tres dimensiones de AR: toma de decisiones, comunicación y libertad de coerción. Los hallazgos muestran una interacción compleja de factores individuales, relacionales y estructurales que influyen en la AR de las adolescentes, con similitudes y singularidades basadas en la situación de escolarización. Los principales obstáculos eran dominio de los padres y parejas en la comunicación y la toma de decisiones, conocimiento limitado de las niñas sobre salud sexual y reproductiva, baja autoeficacia para tomar decisiones y aplicarlas, dependencia financiera y falta de conceptos básicos de la vida. En cambio, las relaciones y escolarización de apoyo surgieron como facilitadores esenciales. En conclusión, a fin de mejorar la AR se necesitan intervenciones multinivel y multifacéticas para abordar los obstáculos a la vez que se aprovechan los facilitadores, por ejemplo: empoderamiento económico del hogar, programas de apoyo a padres y políticas que promuevan el acceso universal a la educación.

## Introduction

Reproductive autonomy, defined as the power to make decisions and exercise control over matters related to contraceptive use, pregnancy, and childbirth^1^, is a key aspect of women’s sexual and reproductive health (SRH). It includes the right to decide whether, when, and how to have children, as well as access to the resources and support needed for informed decision-making. Reproductive autonomy encompasses the interconnected dimensions of decision-making, freedom from coercion, and communication.^1^ Decision-making refers to a woman’s ability to make informed and independent choices regarding her reproductive health. Freedom from coercion means that reproductive decisions are made without force, pressure, or manipulation from partners, family members, institutions, or the state. Communication involves the ability to openly discuss reproductive health matters, which promotes informed choices and mutual understanding within relationships and society.

As a fundamental human right,^2^ reproductive autonomy is rooted in feminist theories,^3^ and serves as a critical indicator of women’s empowerment in society.^4^ It is aligned with the broader concept of personal autonomy, which refers to the capacity for self-governance, independent action, and informed decision-making. (Agich, 1994) Reproductive autonomy is also a key objective of Sustainable Development Goal 5, which seeks to achieve gender equality and to empower all women and girls through the promotion of universal access to SRH and the transformation of unequal power dynamics between men and women.^5^ However, adolescents’ choices are negotiated within social expectations, limited resources, and unequal power relations.^6^ Their reproductive decisions may therefore involve using strategic and covert forms of agency to navigate constraints. Structural vulnerabilities, such as poverty, gender norms, stigma, and limited access to adolescent-friendly SRH services restrict girls’ ability to act on their intentions.^7^ Thus, reproductive autonomy is not only an individual attribute but also a socially and structurally produced capacity, requiring interventions that address both empowerment and the conditions that limit choices.

Gender and power dynamics in heterosexual relationships play a key role in shaping reproductive autonomy.^8–10^ Patriarchal norms and gender inequalities heavily influence reproductive decision-making, often limiting women’s ability to make autonomous choices.^11^ These inequalities hinder open communication with partners and restrict women's ability to make decisions regarding sex and reproduction.^8^ Many women in heterosexual relationships lack the power to refuse sex, which compromises their reproductive autonomy.^12^

In Uganda, reproductive autonomy is largely influenced by restrictive abortion laws, entrenched gendered power relations, poverty, and gaps in SRH services.^13–14^ Many Ugandan women lack economic independence due to unequal access to education, employment, capital, and land for farming.^15–17^ The resultant economic dependence restricts their ability to make choices about contraceptive use, family size, or to leave an abusive relationship.^18^

The structural gender-based inequalities related to reproductive autonomy are strongly correlated with a woman’s age. Younger women, particularly adolescent girls, have lower reproductive autonomy compared to older women.^1^ In response, the Ugandan government has implemented interventions aimed at empowering adolescent girls and enhancing their reproductive autonomy. These include the provision of free primary and secondary education to ensure that adolescents remain in school to acquire knowledge and skills, access SRH information, and improve their future employment prospects for economic independence.^19^ Additionally, the Ministry of Health promotes youth-friendly healthcare services to ensure access to confidential, culturally appropriate, and tailored SRH services that address the specific needs of young people.^20^ However, the effectiveness of these national initiatives is limited by entrenched gender-related socio-cultural and socio-economic inequalities that continue to disadvantage girls.^21^ As a result, low reproductive autonomy among adolescents persists and heightens their vulnerability to adverse SRH outcomes, including sexually transmitted infections such as HIV,^22^ unintended pregnancies,^23^ unsafe abortions, and limited access to modern contraceptives.^24^ These challenges often lead to school dropouts, early marriages, and early childbirth,^25^ further reinforcing the cycle of gender inequality.

Addressing these disparities requires early and targeted interventions to enhance reproductive autonomy among adolescent girls. Such efforts must be grounded in a comprehensive understanding of the contextual and age-specific barriers that adolescent girls face, in order to effectively address their unique challenges while leveraging opportunities for empowerment. Despite the growing recognition of reproductive autonomy as a critical component of adolescent SRH, there remains a dearth of empirical evidence in Uganda, particularly from the experiences of adolescent girls. Few studies have examined how socio-cultural, economic, and relational factors shape the ability of adolescent girls to exercise reproductive autonomy. This study, therefore, seeks to fill this gap by exploring the barriers and facilitators of reproductive autonomy among adolescent girls in western Uganda.

## Methods

### Study design

This study was part of a larger convergent parallel mixed methods study. The aim of the larger study was to advance knowledge on the role of social capital in enhancing the reproductive autonomy of adolescent girls in western Uganda. This qualitative sub-study focused on exploring adolescents’ experiences and perspectives on barriers and facilitators for reproductive autonomy through individual interviews.

### Settings

The survey from which participants for this qualitative component were selected was conducted in Fort Portal city within selected secondary schools and communities.

### Participants

Older adolescent girls (15–19 years), both in-school and out-of-school, were recruited in the study. Participants were purposively selected from 504 respondents who were part of a larger cross-sectional survey, ensuring variation by schooling status, age, and place of residence (urban, peri-urban, and rural). Eligible girls had been or were currently in a sexual relationship and could communicate in Rutoro or English. Participants provided their own telephone contacts or those of close relatives. In-school girls often provided the contacts of their senior women teachers (female teachers designated to handle social and health concerns of female students).

### Procedure

The research team contacted pre-selected participants by phone to schedule the individual interviews. Interviews with in-school participants were conducted at their respective schools, while those with out-of-school participants took place either at their homes or at a nearby public facility. Prior to data collection, all participants were given detailed information about the study and written consent was obtained. For minors, assent was secured in addition to consent from their legal representatives, either parents/primary caregivers at home, or head teachers at school. However, for five out-of-school emancipated minors, including those who were married, pregnant, or living independently, parental/caregiver consent was not obtained. Potential participants who could not be reached after two call attempts made on different days, or those whose phones were switched off (*n* = 9), were excluded from the study. In three cases, the individuals who answered the phone did not recognise the adolescent girls being referred to, suggesting that incorrect phone numbers had been recorded; these were also excluded.

### Data collection

Data collection was carried out between September and November 2024 through individual interviews. These interviews took place in quiet, private settings, either in rooms provided by head teachers at schools, spaces offered by primary caregivers at home, or suitable nearby community facilities when the home environments were not appropriate. Interviews lasted for an average of 54 minutes and they were audio-recorded with the participants’ consent. Two female research assistants, both nurses working in adolescent clinics, conducted the interviews. The assistants were native speakers of the local language and were also fluent in English. The assistants had prior training and experience in conducting interviews with young people from earlier studies with the corresponding author (LGA). The assistants also received a one-day orientation to conduct interviews in this study. The interview guide (Supplemental File 1), which included unstructured questions designed to explore barriers and facilitators across different aspects of reproductive autonomy, namely decision-making, freedom from coercion, and communication, was translated into Rutoro. Although the interviews were primarily conducted in Rutoro, some school-going participants occasionally responded in English. Research assistants also took notes during the interviews to capture key insights, and by the 31st participant no new information was obtained indicating that data saturation had been reached.

### Data management and analysis

Audio files were transcribed verbatim and later translated to English by two postgraduate students of Public Health who were native speakers of Rutoro and fluent in English. The main researcher, LGA, sampled the transcripts and listened to the selected audio to ensure that data was not lost in the transcription and translation process and data analysis commenced.

Data were analysed following the five steps of framework analysis^26^ guided by the dimensions of reproductive autonomy. In the initial step, **familiarisation**, four authors (LGA, SV, EK, JB) immersed themselves in the data by thoroughly reading all transcripts to gain an initial understanding of the participants’ perspectives and key messages. In the second step, **identifying a thematic framework**, we began by open coding eight transcripts. The coding process was primarily inductive, staying as close as possible to the data. In several cases, we employed *in vivo* codes to preserve the participants’ exact words. The open codes were then organised into slightly more abstract inductive codes, which helped us to identify themes. These themes were subsequently categorised under barriers and facilitators for each of the three dimensions of RA: decision-making, communication, and freedom from coercion. In the third step, **Indexing**, LGA systematically applied the developed thematic framework to the remaining data, remaining open to new themes that emerged. NVivo 15 software was used to manage the data at this stage, which facilitated the organisation and tracking of codes, themes, and associated data. In the fourth step, **Charting**, data were organised into a framework matrix using NVivo. The software enabled us to maintain a clear trail of codes, themes, and overarching categories, ensuring consistency and ease of reference throughout the analysis process. In the final step, **Interpreting the data**, we explored typologies and patterns in the data, examining the relationships between themes and overarching categories. This structured approach allowed us to generate a comprehensive report of our findings.

### Ethics

Ethical clearance for the project was obtained from the research and ethics committee of Mbarara University of Science and Technology (MUST-2023-1266 dated 4th January 2024). The study was further approved by Uganda’s National Council for Science and Technology (Ref: SS2627ES dated 22nd August 2024). Administrative clearance was obtained from the City Health Office of Fort Portal City and the head teachers of involved schools. We obtained informed written consent from all participants and assent for minors prior to data collection. We further obtained informed written consent from legal representatives of minors except for the emancipated minors. All discussions were conducted in a private place and were moderated by trained research assistants with over five years of experience working with young girls. Each participant was reimbursed for their time after each session at a rate of US$ 6.6. Audio files and transcripts were anonymised with assigned numbers (#1 to #31) and secured by password in the computer of the main researcher.

## Findings

### Characteristics of participants

In total, 31 participants were interviewed (Table 1), with a mean age of 18.16 years (range 15–19 years). The majority (74%) were aged 18 years or older. A substantial proportion (65%) were out of school and primarily living in rural areas. Only a small fraction (10%) of the participants were married.

**Table 1.** Characteristics of participants

Characteristic	Frequency (*n*)	Percentage (%)
Age in years		
Mean (range) 18.16 (15–19)		
<18	08	26
≥18	23	74
Schooling status		
In-school	11	35
Out-of-school	20	65
Residence		
Rural	18	58
Urban	13	42
Marital status		
Single	28	90
Married	03	10

Seven interrelated themes were identified, reporting both barriers and facilitators of reproductive autonomy, with decision-making emerging as a central thread across all themes. The themes were organised into three overarching domains, deductively derived from the reproductive autonomy scale. Across themes, participants’ narratives reflected both shared experiences and notable differences by schooling status. The themes are presented in an order that captures the scope and depth of participants’ narratives.

### Decision-making domain

This category includes themes reporting constraints and enablers for participants to make autonomous and informed decisions regarding their sexual and reproductive health.

#### Self-efficacy

While both in-school and out-of-school participants described limited self-efficacy, in-school adolescents more often linked decision-making to school-based knowledge and future aspirations, whereas out-of-school adolescents more frequently described decision-making within contexts of early union, reduced access to information, and fewer protective structures. Participants frequently reported becoming pregnant inadvertently, citing a lack of information to make informed choices, such as abstaining from sex or using contraceptives, as the main contributing factors. The quote below illustrates the unintended occurrence of pregnancy.

*“About the pregnancy, I didn’t intend it, and it was undesired, but it happened, after understanding that I was pregnant we decide that we should stay together.”* (#12, 15-year-old, out of school)

In some accounts, the narratives revealed lapses in contraceptive use among participants leading to pregnancies. Additionally, personal compromises that contributed to suboptimal decision-making were noted by many. For instance, in the quotes below, one 18-year-old participant reflected on being “blinded by love” when she chose to engage in unprotected sex with her partner, while a 17-year-old reported fearing the consequences of declining her partner’s romantic advances.

*“When you are in love, you can listen and make a wrong decision because you are blinded by love. So, you can do what he tells you to do because you are out of control.”* (#3, 18-year-old, in-school)

*“When he wrote a paper asking me for love, I feared to deny him because I knew he would go around the village talking bad things about me. So I accepted, but deep in my mind, I was thinking I will not stay with him.”* (#7, 17-year-old, out of school)

Some strategies to enhance capacity to make informed and appropriate decisions regarding SRH were reported. Several in-school participants emphasised that staying in school was empowering, as it provided them with knowledge on how to prevent unintended pregnancies and sexually transmitted infections. This perspective was less prominent among out-of-school participants, who more often described learning through experience, peers, or partners, with fewer formal opportunities for SRH guidance. Others reported carefully evaluating potential sexual partners before getting into relationships, demonstrating a deliberate approach to partner selection as typified in the quote below.

*“Before deciding to love him, I made research about him to know who he was from his friends and where he comes from. I found out that he was a good boy who is faithful, who gives his religion and work time other than engaging in peer groups.”* (#2, 19-year-old, in-school)

#### Family and partner involvement

Both support and control by family members and partners were reported in decision making. Out-of-school participants frequently described decisions being shaped by partners within cohabiting or marital arrangements, while in-school participants reported negotiating decisions within parental oversight and schooling expectations. In the quote below, a 19-year-old in-school participant described involving a trusted family member to help assess the suitability of a potential partner before making a decision.

*“My elder sister was involved in that decision [accepting a partner], that sister of mine I am so comfortable with her, I normally share with her my secrets, and so, actually before going into relationship, I had already talked to her, and she was like oh that boy is kinder this and this I like him just because this and this. The day when he initially proposed to me that’s when I became so much excited and I of course had to share the information with my sister and that’s how she came to know.”* (#31, 19-year-old, in-school)

Some described experiences of subtle pressures from parents to conform to parents’ SRH decisions. In situations of disagreement, many reported complying out of respect, dependence, or fear. Some out-of-school participants also recounted instances of direct control by partners over SRH decisions. Notably, in several cases, sexual partners were described as the primary decision-makers, particularly among married adolescents, given their role as key providers. A few participants reported that their partners actively prevented them from using family planning methods as illustrated below.

*“Since he was used to ‘safe days’, he would study them while at school, it was easy for him to use safe days method and he stopped me from using condoms and pills. I hear sometimes that safe days are risky and are not 100 percent, so it was hard on my side because I feared to get pregnant.”* (#20, 18-year-old, in-school)

#### Socio-economic situation

This theme illustrates how poverty, limited education, unemployment, and financial dependence undermined participants’ abilities to make informed and autonomous SRH decisions and measures they employed to remedy such inadequacies. Socio-economic vulnerabilities emerged across multiple levels of participants’ socio-ecological context. Although economic hardship was reported across both groups, out-of-school participants more consistently described poverty as directly shaping relationship choices, cohabitation, and reliance on partners for survival needs, while in-school participants presented school-based vulnerabilities such as dependence on support for fees and meals. Many girls reported lacking a stable source of income while desiring material items such as fashionable clothing and mobile phones, which led some to seek sexual relationships for financial support. This perspective is illustrated by the quote below from a 19-year-old out-of-school girl.

*“There are things that bring us to make that kind of decision we as girls. Now for me for example I do not have my parents. I got challenges on the things that I needed and could not afford to have them and at home when my parents died all of us were still very young and unable and the people who the parents had left to take care of us could not take care and that was the reason why I had to get a boyfriend because he could help me in certain things that I did not have, I could tell him that I don’t have soap he give me or I tell him I have no clothes and gives me, that’s what led me, I don’t have all those kinds of needs.”* (#30, 19-year-old, out of school)

Several out-of-school participants lamented severe household economic hardship and deprivation, which prompted them to elope with sexual partners. They described receiving material inducements such as money or mobile phones from partners, which shaped their decisions to enter relationships, engage in sexual activity, or cohabit. A few participants further reported being lured into sexual relationships with teachers in exchange for academic favours, free meals, or financial assistance at school as illustrated below.

*“How I got to accept him was because my mother, who is a single mother for the four of us, could not manage to buy for us things at home and other socialistic materials. So, the guy used to provide, even would pay lunch, and one time he wanted to pay school fees but I feared to tell my mother.”* (#17, 16-year-old, in-school)

To address socio-economic vulnerabilities, some out-of-school participants reported seeking employment as a way to achieve financial independence and mitigate the risk of economic hardship that could undermine their autonomy.

*“The support that now I need is to be having a business and am working but not working for others* *but you have your own business. That way you can make your own decisions, not your boss to ask you for sex because you are working for him.”* (#24, 19-year-old, out of school)

### Communication domain

This category presents themes in which participants presented obstacles and enablers to open and honest discussions about SRH with people around them.

#### Dialogue and guidance

Prominently reported was the concealment of information by participants regarding SRH. Many shared that they did not discuss with their parents issues such as contraceptive use or being in a sexual relationship, largely due to fear of parental reactions, particularly among those still in school, as well as the absence of a culturally acceptable or applicable way to address such a “sensitive” topic.

*“No, me I have never talked about those things [SRH matters] with them [parents] I was even fearing to talk about it. Well, I didn’t even think she can counsel me, I thought she could say my daughter now have you started getting spoilt.”* (#8, 17-year-old, in-school)

Several participants also noted that their parents lacked time or willingness to engage in such conversations. For some, parents were described as dominating SRH discussions, limiting opportunities for open dialogue. Some reported disregarding their parents’ guidance due to temptations to engage in sexual activity, driven by the socio-economic vulnerabilities outlined in the preceding theme.

Additionally reported was the lack of “true friends” that participants could trust and confide in about sexual matters to seek guidance. One out-of-school participant lamented the erosion of a community tradition where elders would regularly offer advice and mentorship to adolescent girls on such issues. Also, many in-school participants reported the absence of structured SRH education and guidance at school. They expressed that such programmes would equip them with knowledge to empower them make informed choices.

*“Some schools have no guidance and counselling. This means that a girl will decide on her own. This brings about making many wrong choices because of lack of awareness. Even our parents are not giving us time to talk to us.”* (#13, 18-year-old, in-school)

Participants described ways they navigated and enhanced communication to achieve better SRH outcomes, as well as suggestions for further improvement. Some mentioned having supportive friends with whom they could openly discuss SRH issues and receive helpful guidance. For others, particularly those out of school, open, honest, and free communication with parents about SRH matters was seen as a crucial factor enabling communication autonomy.

*“ … Parents should also be free with their girls and tell them that if you do this and this you will end up getting this and this.”* (#5, 19-year-old, out of school)

*“Parents should be able to tell their children facts like when you start a relationship with a boy you might end up getting pregnant, acquiring deadly diseases like HIV. So when a girl is going to sleep with a boy, she will definitely remember those words and will tell the boy to use any safety measure so that nothing bad can happen.”* (#15, 19-year-old, out of school)

One participant suggested the idea of consulting and listening to community elders, who were seen as possessing greater knowledge and experience regarding SRH matters. Additionally, many emphasised the need for structured SRH education in schools, with experts invited to engage adolescent girls on these topics.

*“They [teachers] invite should invite people who know these things [SRH issues] to guide us as adolescent girls by showing us the right path. For example, they can tell us the dangers of unprotected sex.”* (#11, 18-year-old, in-school)

#### Myths and misinformation

Misinformation about SRH was widely reported with many participants noting that much of their knowledge came from less trusted sources such as peers and social media. Common myths included beliefs that contraceptives cause infertility, lead to excessive weight gain, and increase the risk of cancer. The quote from participant #27 below exemplifies such misconceptions. This challenge was reported by both in-school and out-of-school participants, although out-of-school participants more often described limited access to alternative trusted sources such as teachers or school-based programmes. The misconceptions influenced the participants’ decisions regarding contraceptive use.

*“They can definitely say that it would be difficult for her to produce in future because of the side effects family planning methods come with. They can say that she may even get diseases and even spoil other girls in the village.”* (#27, 19-year-old, in school)

### Freedom from coercion domain

This category presents themes in which participants reported pressures, manipulation, force or intimidation that they experienced regarding SRH decision-making and enablers to overcome them. Out-of-school adolescents often described early marriage, pregnancy-related exclusion from the parental home, and increased dependence on partners, which reduced their ability to resist coercion. In contrast, in-school participants more often framed coercion risks within school and community environments, including fear of stigma, relationship pressures, and exposure to intoxicated partners.

#### Social pressures and sexual abuse

Several participants described the influence of prevailing cultural and religious norms in their communities that constrain their personal autonomy. The two quotes below from participants #9 and #23 exemplify these influences.

*“In our cultures, you find a girl is supposed to get married at that age, immediately when you turn 18 years you need to get married so sometimes cultures also make us make wrong decisions.”* (#9, 19-year-old, in-school)

A number of them highlighted a widespread practice of denying girls access to education, rooted in cultural expectations that girls should marry upon reaching puberty.

*“Culturally, you find a girl is forced into marriage at early age of course that has to be changed. By the way, a girl should be given freedom to do what she feels like in terms of education. In some cultures, girls are not supported in terms of education because of this thing that a girl’s office is marriage and is all about cooking, housewives, so that has to be changed, also a girl has a right to education, so they must be freed to education that they improved on their life styles.”* (#23, 18-year-old out of school)

Among out-of-school adolescents, it was frequently reported that those who became pregnant were compelled to leave their parental homes and reside with their partners, as premarital pregnancy is regarded as a cultural and religious taboo.

It was also noted in a few cases that parents actively pressured their adolescent daughters into forming relationships with men, motivated by the expectation of receiving financial benefits. This practice was reported by an out-of-school participant. Conversely, some described experiences of parental overprotection, wherein parents restricted their daughters’ interactions with others, particularly men, thereby limiting their social engagement and autonomy as presented by participant #5 in the quote below.

*“Again, my family are so strict in a way that when they don’t want me and my sisters near boys, or even walking with them, which I don’t see with other girls.”* (#5, 19-year-old out of school)

A few participants reported experiences of forced sexual intercourse perpetrated by their partners. Such incidents were commonly associated with partners who were intoxicated by alcohol or other illicit substances as illustrated in the quote below.

*“No, he called me and I came where he was asking me to come as fast as possible, and he would not spend long with me. The second time I slept with him, he caught me by force and it's when he was going to [name of a place]. This happened whenever I would find him drunk.”* (#1, 17-year-old in school)

Notably, one participant recounted initiating contraceptive use as a proactive measure to prevent unintended pregnancy that could arise from such forced sex.

#### SRH laws and services

Many participants described the healthcare system as unfriendly when seeking SRH services. They reported encountering healthcare workers who exhibited negative attitudes, characterised by rudeness, judgmental behaviour, and verbal abuse. These interactions fostered feelings of stigma and rejection. Both in-school and out-of-school adolescents described these barriers, though out-of-school participants more often reported seeking SRH services independently, including antenatal care, after leaving school or entering unions. One participant recounted a situation in which a healthcare worker questioned her about her sexual partner during the antenatal care visit. This left her wondering if she had no right to seek care without the partner as illustrated below.

*“ … like in the hospital, the first time I visited hospital for antenatal they asked where my man was, so you find so many girls that come for antenatal have no answer to that question, and are not sure of the ones that impregnated them. They should change because they say things that can make you get annoyed because being young doesn’t mean you can’t do what you want.”* (#5, 19-year-old, out of school)

Good interpersonal relationships and law enforcement were noted as key enablers of freedom from coercion in SRH matters. Many emphasised that positive, supportive relationships with parents and partners created a safe environment where they felt listened to, understood, and guided, rather than forced to act against their will. Additionally, participants highlighted the importance of legal protections, noting that the enforcement of relevant laws, such as those addressing rape and forced marriage, was crucial in safeguarding their sexual and reproductive rights.

## Discussion

We explored barriers and facilitators of reproductive autonomy for adolescent girls in western Uganda, drawing on their experiences and perspectives. This is the first study to examine this topic among adolescents in Uganda. The findings reveal the interplay of individual, relational, and structural factors shaping girls’ reproductive autonomy across decision-making, communication, and freedom from coercion. While both in-school and out-of-school adolescents described similar constraints, the pathways through which these barriers operated often differed. A central finding was limited decision-making capacity, characterised by low confidence, inadequate knowledge, and external control by partners and family. This finding reflects barriers in the decision-making domain of reproductive autonomy, where girls lacked the information, self-efficacy, and power needed to make informed choices aligned with their reproductive intentions. The findings further portray age-related constraints on autonomy and the need for empowerment through education. Consistent with earlier studies,^1,27^ we found that adolescents’ SRH decisions are shaped by power imbalances and gender norms prioritising male or parental authority. These inequalities highlight the need for interventions that equip girls with negotiation skills and promote equitable norms. Schools offer an ideal setting for such programmes due to their structured environment and wide reach. However, out-of-school adolescents may not benefit from school-based interventions and thus there is a need for community-based platforms that reach girls outside formal education. Participants’ narratives showed how inadequate knowledge and low confidence undermined agency, often resulting in decisions misaligned with their reproductive intentions. Many, especially those married or financially dependent, described partners as primary decision-makers about contraception, revealing the pervasive influence of patriarchal structures.^28–30^ This influence was more prominent among out-of-school participants. Such partner dominance represents reduced reproductive autonomy through limited control over contraceptive decision-making and diminished ability to act on personal preferences. The finding aligns with broader evidence from sub-Saharan Africa showing that male preferences often constrain girls’ contraceptive choices.^31,32^ This partner dominance downplays efforts to promote individual choice and calls for strategies engaging male partners and families to support girls’ autonomy, a priority for future policy and research.

Socio-economic vulnerability emerged as a critical structural barrier among participants. Engaging in sexual relationships was sometimes a survival strategy, reflecting unmet material needs and limited parental support. Structural constraints shape reproductive autonomy by limiting the actual choices available to girls, thereby reducing their ability to make voluntary and self-directed reproductive decisions. Although both in-school and out-of-school adolescents reported poverty as a barrier, the financial needs and vulnerabilities due to unmet needs differed. This finding supports literature on transactional sex among adolescents in resource-constrained contexts.^33^. Poverty, young age, and gender inequality intersected to reinforce girls’ vulnerability. Addressing this requires policy measures to strengthen household economic capacity and provision of all school basic requirements, which could reduce girls’ risk of sexual exploitation driven by financial insecurity.

The lack of open dialogue with parents and trusted adults deprived girls of accurate SRH information, compelling them to rely on peers and media, who often provided uncensored and wrong information. This barrier in the communication domain of reproductive autonomy limited opportunities for guidance and discussion, reducing girls’ capacity to seek support, clarify misinformation, and negotiate SRH choices. SRH remains a sensitive topic among families within the setting of this study,^34^ similar to findings from other contexts where communication taboos persist.^35^ Yet, given adolescents’ high need for information,^36^ alternative sources are sought, whose reliability may be questionable. Fear of parental dominance and negative reactions further hindered communication autonomy, limiting opportunities for guidance. Community interventions that build parents’ capacity to engage in open, age-appropriate dialogue while supporting girls’ autonomy are essential. These could take the form of parent–adolescent communication support programmes as reported in a review by Gavin et al.^37^

Cultural and religious norms promoting early marriage and stigmatising premarital pregnancy constrained girls’ reproductive choices, and at times compelled them into early relationships or premature childbearing roles. These norms restrict freedom from coercion, as they create social pressure and sanctions that limit girls’ ability to make voluntary reproductive decisions. While Uganda’s laws prohibit child marriage, these unions persist, often protected by cultural norms and revealed only by whistle-blowers.^38,39^ Sustained community sensitisation is critical to shift attitudes and reduce early marriage. Out-of-school participants more frequently described early marriage, pregnancy-related exclusion from the parental home, and increased dependence on partners, which further reduced their ability to negotiate SRH decisions. Some participants also reported coercion and forced sex, stressing the need for legal protection and services to address gender-based violence. Negative experiences with health providers, described as judgmental or unsupportive, further discouraged girls from seeking help, consistent with findings from another study.^40^ Such experiences undermine reproductive autonomy by limiting access to supportive services required for girls to act on their reproductive intentions and seek protection when autonomy is threatened. The limited awareness of legal rights and lack of adolescent-friendly services reduce the capacity of the girls to pursue justice, while stigma around adolescent sexuality adds further barriers. Without deliberate efforts to create safe, informed, and non-judgmental environments in families and health systems, legal protections remain inaccessible to this age-group. To address adolescents’ negative experiences with healthcare providers, the delivery of adolescent-responsive and non-judgmental SRH services should be strengthened. The strengthening of service delivery may include in-service training of healthcare workers in respectful communication, confidentiality, and rights-based care, alongside supportive supervision to reduce stigma and discriminatory attitudes.

Despite the aforementioned barriers, some facilitators emerged. Supportive relationships with parents, siblings, and peers enabled some girls to access information and make informed choices. These facilitators strengthen reproductive autonomy by enhancing girls’ decision-making and communication autonomy, while also offering protection against coercion. Reliance on friends and sisters as trusted guides supports earlier findings on the importance of peer influence.^41^ However, unequal access to informed, supportive peers, especially among vulnerable girls, calls for formal support structures. Schools can provide safe spaces, accurate SRH education, and mentorship to complement informal networks and promote equitable access to guidance. In-school participants viewed schooling as empowering, offering knowledge and protective structures that delayed sexual initiation and improved contraceptive awareness, supporting evidence linking education to delayed sexual debut and increased reproductive agency.^42^ For instance, one of the largest comprehensive sexuality education programmes in Uganda, *The World Starts With Me*, was found to be effective in delaying sexual debut, improving safe sex practices, and preventing coercive sex among school-going adolescent girls.^43^ Structured, expert-led SRH education within schools can thus advance adolescent girls’ reproductive autonomy. Beyond formal instruction, schools also serve as platforms for peer mentorship and future-oriented engagement, reducing early sexual involvement. While Uganda provides free basic education, barriers such as school-related gender-based violence, early pregnancy, sociocultural expectations, and household duties still affect attendance and completion. These challenges merit further study and targeted interventions to strengthen girls’ sustained engagement in school, ultimately supporting their reproductive autonomy and broader well-being. In contrast, out-of-school adolescents more often described the need for livelihood opportunities and community mentorship as key enablers of autonomy, suggesting that economic empowerment and outreach SRH education may be especially relevant for this group.

### Implications for practice and policy

The findings support the need for multi-level interventions to promote reproductive autonomy among adolescent girls in Uganda. These interventions can be implemented within existing national frameworks such as the National Sexuality Education Framework (2018), the National Adolescent Health Policy Guidelines and Service Standards, and school-based and youth-friendly programmes including peer education initiatives.^44^ Strengthening comprehensive sexuality education in schools is important in providing adolescents with accurate SRH information, communication skills, and decision-making abilities. Evidence from the decade-long “World Starts With Me” programme in Uganda showed that comprehensive sexuality education promoted reproductive autonomy among school-going adolescents.^43^ These findings are consistent with the current study, where access to trusted information and supportive guidance improved girls’ confidence in making reproductive decisions. Our findings therefore support comprehensive sexuality education approaches that address not only biological information, but also gender norms, consent, communication, and relationship power dynamics. The findings further highlight the importance of keeping girls in school through policies that support universal education and school re-entry for pregnant adolescents. In addition, community-based interventions should engage parents, male partners, teachers, and community leaders to address harmful social norms and create supportive environments that enhance adolescent girls’ reproductive autonomy.

### Study strengths and limitations

The study provides valuable nuanced insights into what hinders or enables the reproductive autonomy of adolescent girls. The application of the three dimensions of reproductive autonomy enhanced the study’s rigour, ensuring a structured interpretation of participants’ experiences and perspectives. However, this study had some limitations, such as potential recall bias, since participants might not have accurately remembered their experiences, and social desirability bias, which could have shaped how they responded. To minimise social desirability bias, interviews were conducted privately in a confidential, non-judgmental setting. Trained interviewers used neutral, open-ended questions and reassured participants that there were no right or wrong answers. Additionally, the study focused solely on adolescent girls without incorporating perspectives from parents, sexual partners, teachers, or health workers, limiting a holistic understanding of barriers and facilitators.

## Conclusion

This study brings to fore the complex interaction of individual, relational, and structural factors influencing reproductive autonomy among adolescent girls in western Uganda. The findings reveal key limitations in decision-making capacity, stemming from inadequate knowledge, low self-efficacy, and external control by partners and family members. These barriers are further compounded by socio-economic vulnerability, restrictive gender norms, limited parental communication, and weak institutional support. Facilitators such as peer and familial support, school attendance, and structured sexuality education appear as pathways to enhance adolescents’ reproductive autonomy. Strengthening implementation of existing national frameworks, such as the National Sexuality Education Framework and the Adolescent Health Policy guidelines could improve adolescents’ access to accurate SRH information and adolescent-friendly healthcare services. There is also need to strengthen school retention strategies for girls, expand community-based and peer-led sexuality education programmes, and promote interventions that engage parents, male partners, cultural leaders, and communities in addressing harmful gender norms and improving communication around SRH. Additionally, socio-economic empowerment initiatives targeting vulnerable adolescent girls may help reduce dependence on partners and improve their ability to make independent reproductive decisions.

## Provenance statement

This article was not commissioned and went through external peer review.
